# Supplementary material for: Knockout of zebrafish desmin genes does not cause skeletal muscle degeneration but alters calcium flux
Source: Sci Rep. 2021 Apr 5;11:7505. doi: 10.1038/s41598-021-86974-w (PMC8021586; doi:10.1038/s41598-021-86974-w)
Supplement: Supplementary file 2 — Supplementary Table S1. [file 41598_2021_86974_MOESM2_ESM.pdf]

SUPPLEMENTARY TABLE

Knockout of zebrafish desmin genes does not cause skeletal muscle degeneration but alters calcium flux

Gulsum Kayman Kurekci, Ecem Kural Mangit, Cansu Koyunlar, Seyda Unsal, Berk Saglam, Bora Ergin, Merve Gizer, Ismail Uyanik, Niloufar Boustanabadimaralan Düz, Petek Korkusuz, Beril Talim, Nuhan Purali, Simon M. Hughes, Pervin R. Dincer.

Table S1. List of primer sequences used in for synthesis of ISH probes, genotyping and qPCR experiments.

	**Forward primer sequence**	**Reverse primer sequence**
desma antisense probe	5'-TACATCGAGAAGGTGCGCTT-3'	5'-GGATCCATTAACCCTCACTAA AGGGAATTGTCTCCATGCGTCAT CCA-3'
desmb antisense probe	5'-AATGACCGCTTCGCCAACTA-3'	5'-GGATCCATTAACCCTCACTAA AGGGAACCTCTCCATCACGTGTC TCG-3'
desma sense probe	5'-TAATACGACTCACTATAGGGA GATACATCGAGAAGGTGCGCTT-3'	5'-TTGTCTCCATGCGTCATCCA-3'
desmb sense probe	5'-TAATACGACTCACTATAGGGAG AAATGACCGCTTCGCCAACTA-3'	5'-CCTCTCCATCACGTGTCTCG-3'
desma genotyping primers	5’-CGCACGGTGGCTGGAAAA-3’	5’-CACGGTTCCTCAGGTTCCTC-3’
desmb genotyping primers	5’-GTCATCCACTTCTTCTCCTGG-3’	5’-GTGCGAGTGTGGAGAAACTC3’
desma qPCR primers	5’-GCTGCCAAGAATATCAGCGA-3’	5’-TGCCTCCTCAGAGACTCATT-3’
desmb qPCR primers	5’-ATGCAAGAGACCCAAGTCCA-3’	5’-TCTTGCTCACAGCCTGGTTA-3’
actb1 qPCR primers	5’-TCTTCCAGCCTTCCTTCCTG-3'	5’-TGTGTTGGCATACAGGTCCT-3’
vim qPCR primers	5’-ACCTAGGGGAGGACATCGAG-3’	5’-CGAGCCAGAGAGGCGTTATC-3’
rpl13a qPCR primers	5'-TCTGGAGGACTGTAAGAGGTATGC-3'	5'-AGACGCACAATCTTGAGAGCAG-3'
